# Influence of colors in distractor videos on the recording of cortical auditory evoked potentials

**DOI:** 10.1590/2317-1782/e20250352en

**Published:** 2026-07-27

**Authors:** Jamyle Rodrigues Luis, Pedro de Lemos Menezes, Carlos Henrique Alves Batista, Kelly Cristina Lira de Andrade

**Affiliations:** 1 Departamento de Fonoaudiologia, Universidade Estadual de Ciências da Saúde de Alagoas – UNCISAL - Maceió (AL), Brasil.

**Keywords:** Electrophysiology, Auditory Evoked Potentials, Speech, Audiology, Speech Therapy

## Abstract

**Purpose:**

To compare the morphological differences in CAEP obtained with black-and-white versus colored distractor videos, and to verify whether the black-and-white format reduces response variability.

**Methods:**

This cross-sectional analytical study, approved by an ethics committee (protocol nº 3.477.022), included 30 adults aged 18–35 years with normal hearing. Participants underwent CAEP recordings using /ba/ and /da/ stimuli under two conditions: silent black-and-white and silent colored videos. Latencies, amplitudes, and interlatency intervals were analyzed using paired t-tests and Levene’s test (p < 0.05).

**Results:**

Colored videos resulted in longer latencies for P1 and P2 in both ears, reduced P1 amplitude in the right ear, and increased P2 amplitude bilaterally. The P1-N1 interval was greater with black-and-white videos, while the N1-P2 interval increased with colored videos in the left ear.

**Conclusion:**

The use of colored videos influences CAEP morphology and variability and should be carefully considered in clinical and research contexts.

## INTRODUCTION

Cortical Auditory Evoked Potentials (CAEP) allow the assessment of the entire auditory system from the brainstem to the auditory cortex. CAEP recordings can be obtained through different types of stimuli, such as *tone burst* and/or more complex stimuli such as speech^(1)^.

CAEP has proven to be an effective method in investigating the arrival of sound information to the auditory cortex, especially the auditory processing of information, since the capture of these potentials reflects the cortical activity involved in the brain's discrimination, integration and attention skills^(2,3)^.

The responses generated by CAEP correspond to a series of peaks with negative (N) and positive (P) polarities, forming a P1-N1-P2 wave complex^(4)^. These potentials are analyzed regarding their latency and amplitude, considered as the most important parameters to be analyzed^(5)^.

The P1 component reflects the coding of the acoustic characteristics of sound, such as frequency and time^(6)^. The N1 component is considered a marker of auditory cortical activity, which represents the decoding of acoustic characteristics^(7)^. The P2 wave, more precisely its amplitude, is related to auditory discrimination, with an improvement in its amplitude after acoustically controlled auditory training^(8)^.

To accurately capture and record the waveforms, the professional must control various variables, including the interference from undesirable electrical artifacts. It is recommended that such interference not exceed 10% of the total number of stimuli to prevent low-quality recordings, which can render the analysis of the waveforms unfeasible^(9)^. Another important parameter to be controlled is the filter, an essential resource for properly capturing the electrical signal by improving noise interference^(10)^. Another necessary precaution is adequate cleaning of the skin to reduce contact impedance and enhance the capture of responses^(11)^. In addition, the state of sleep and wakefulness must also be controlled, as this variable can result in low reproducibility of the waves, with lower amplitudes^(12)^.

For managing sleep and wakefulness during examinations, using visual aids proves highly effective in maintaining patient alertness. Clinically, it is customary to display silent videos to prevent interference with the acoustic stimuli delivered through the transducer. Moreover, opting for black and white visuals is a common practice during CAEP assessments; recommendations for the specific use of such visual aids remain unclear, though^(13)^.

Studies that compared the differences in electroencephalographic waves after the inference of a visual evoked potential using black and white and color stimuli determined that visual stimuli with colors demand greater brain activation compared to the presentation of visual stimuli in black and white^(14^**^,^**^15)^. The visualized colors depend on properties of the nervous system so that light detection occurs through the activity of specialized cells that transform light energy into a neural response. These cells are the photoreceptors of the retina, the cones and rods, and their ability to detect light varies across the electromagnetic spectrum, from red to violet. Daytime vision, which allows us to see colors, is mediated by cones, whereas nighttime vision is facilitated by rods^(16,17)^.

Therefore, the present study aims to answer the following guiding question: What are the morphological differences in CAEP when using black and white and color distractor videos? It aims to assess the morphological differences in CAEP traces when using black and white and color distractor videos, and to identify whether the presentation of black and white distractor videos results in less response variability.

## METHODS

This is an observational, cross-sectional, analytical study, conducted based on recommendations of the *STrengthening the Reporting of OBservational studies in Epidemiology* – STROBE. The research was carried out at the Hearing and Technology Laboratory – LATEC at Universidade Estadual de Ciências da Saúde de Alagoas – UNCISAL, submitted and approved by the research ethics committee of the Institution, under no. 3,477,022.

The sampling technique was non-probabilistic and for convenience. The study sample consisted of 30 adult subjects, without hearing impairment, sized from the sample calculation for comparison of two means, in which the alpha value was equal to 0.05, and beta equal to 0.1, the standard deviation was equal to 7.8 ms (largest standard deviation observed in the pilot test of the present study) and the difference between the groups was 9 ms.

The following inclusion criteria were adopted: age between 18 and 35 years, both sexes, tonal auditory thresholds up to 25 dB^(18)^; type A tympanometric curve and the presence of both ipsilateral and contralateral stapedial reflexes; intact auditory pathways up to the brainstem, as assessed through Brainstem Auditory Evoked Potential (BAEP) using a click stimulus and a score of 26 or higher in the Montreal Cognitive Assessment (MoCA), which indicates no cognitive deficits. Participants with a history of alterations in the external and/or middle ear, ear surgeries and presence of cognitive or behavioral changes were excluded from the study. Initially, the volunteers were given an explanation of the procedures that would be performed and the purpose of the study. After acceptance, the volunteers were instructed to sign the Free and Informed Consent Form.

As a pre-collection measure, the following procedures were performed: 1) Inspection of the external auditory canal using a Heine® mini 3000 model otoscope to exclude possible alterations in the external ear and tympanic membrane; 2) Application of the MoCa test to exclude possible mild cognitive impairments, with a score of 26 or higher considered within the normal range^(19)^; 3) Immittance testing using the Interacoustics® AT 235 middle ear analyzer, in which the ears were assessed separately at frequencies of 500, 1000, 2000 and 4000 Hz; 4) Air conduction pure tone audiometry at frequencies from 250 to 8000 Hz, including interoctave frequencies of 3000 and 6000 Hz, using supra-aural transducers (model DD45), on an Interacustics – AD 626 audiometer, within a Vibrasom® acoustic booth, according to the recommendations S3.1 of the American National Standard Institute - ANSI.

The psychoacoustic method of investigating hearing thresholds was used through the descending technique, with 10 dB intervals, and ascending technique, with 5 dB intervals to confirm the responses; 5) BAEP using a click stimulus and duration of 100 µ sec, recording window of 10 ms, speed of 21.1/s, low-pass filter of 3000 Hz and high-pass filter of 100 Hz of the EEG and gain of 100.0 K. One thousand sweeps, with rarefaction polarity confirmed by condensation were performed for each stimulation at an intensity was 80 dB HL. The skin was cleaned with alcohol and abrasive paste, followed by the electrode placement, configured as follows: two negative polarity electrodes positioned on the mastoid region (M1 and M2); a positive polarity electrode placed at the Cz position; and the ground electrode positioned on the lower region of the forehead (Fpz). During the analysis of the traces, attention was given to the latencies, amplitudes, morphology and reproducibility of waves I, III and V, as well as the interpeak intervals I-III, III-V and IV. Those results with an increase in absolute latency above two standard deviations and/or absence of any of the peaks were considered altered^(20)^. Navigator PRO - Biologic® was used.

Participants were then directed to perform the collection procedure, which consisted of performing the CAEP with speech stimuli recorded ipsilaterally to the stimulus. The stimuli were presented, via insert earphones (EAR-phones 3A), monaurally, to the right and left ears and randomized order of presentation of the auditory stimuli between the ears. The duration of the examination was approximately 40 minutes per participant. Navigator PRO - Biologic® was also used. The speech stimuli (synthetic syllables /ba/ and /da/, available for the Biologic system) were presented at a speed of 0.7 stimuli/second, with a 40 ms duration, with 50 deviant stimuli and 250 standard stimuli (*oddball paradigm*) at an intensity of 80 dB HL. In each sweep, the percentage of artifacts accepted was less than 10% of the total stimuli. Two scans were performed and the analysis was conducted on the resulting trace of their weighted sums for each of the test conditions. The oddball paradigm was chosen because it is a well-established and reliable method for eliciting CAEP. Although deviance detection was not the focus of this study, the oddball structure provides a controlled and consistent auditory presentation that enhances the signal-to-noise ratio and supports the extraction of stable P1–N1–P2 responses.

To capture the CAEP responses, participants were positioned in a reclining chair in an acoustically treated room and instructed to remain relaxed and awake. The electrode region was properly prepared with NUPREP abrasive paste, and then the disc-type electrodes were placed with the aid of a conductive paste, with placement following the International 10-20 System, in the following positions: positive electrode at Cz (vertex); negative electrode on M2 (right mastoid); negative electrode on M1 (left mastoid); ground electrode at Fpz (forehead).

During the procedure, participants were instructed to pay attention to a 40-minute Charles Chaplin film that was presented via a smartphone in two versions: in color and black and white, both without sound, with a randomized order of presentation. Although luminance, brightness, and contrast were not objectively measured or equalized between the videos, both stimuli were obtained from the same source, presented at the same size and duration, and displayed under identical ambient lighting and equipment settings. The visual stimuli were presented in their original format (mp4), with a resolution of 1280×720, a file size of 324 MB, and a total duration of 30 min 54 s. Additionally, no visual acuity screening was conducted prior to participation.

The CAEPs were recorded by a trained examiner and the marking of the P1, N1 and P2 waves was performed by at least two researchers experienced in electrophysiology. When the trace was difficult to analyze, without agreement on the marking, all professionals involved in the study discussed it until consensus was considered. It is noteworthy that the researchers responsible for marking the waves were blinded to the recording condition. Disagreements between evaluators were minimal and were not formally quantified. All instances of divergence were discussed and resolved by consensus.

The marking and identification of each wave was performed manually to assess its morphological characteristics and relevant temporal aspects using the Smart Tools EP tool, 2.3^(21)^.

### Statistical analysis

Statistical analysis was performed using the Statistical application Package for the Social Sciences, version 28.0 for masOS version Sonoma 14.5. To describe the data, tabular and graphical presentation of means, standard deviations and confidence intervals were used. Initially, the sample was assessed to observe its adherence to the normal distribution using the Shapiro-Wilk test.

The comparative analysis of the traces using color and black and white video was performed using the T-test for paired samples*.* The assessed parameters were latencies, interlatency intervals, and amplitudes.

To verify variance homogeneity between the two types of visual stimuli, the Levene Test was applied. Analyses were conducted for each ear and each CAEP wave component, comparing the variances of latencies and amplitudes between the black and white and color conditions. In addition, standard deviations were calculated for each stimulus condition.

Differences were considered significant for p-values less than 0.05.

## RESULTS

Of the 30 participants, 13 (43.33%) were male and 17 (56.67%) were female. The age range was between 20 and 34 years, with a mean age of 25.5 ± 3.99 years; 16 (53.33%) had completed higher education and 14 (46.67%) were completing higher education. Two (6.67%) participants were left-handed and 28 (93.33%) were right-handed.

Table 1 shows the descriptive analysis (mean, standard deviation and upper and lower 95% confidence interval) and analytical analysis of the latency, amplitude and interlatency interval values of the P1, N1 and P2 waves for the CAEP test conditions.

**Table 1:** Descriptive and analytical analysis of the latency, amplitude and interlatency interval values of the P1, N1 and P2 waves of the thirty participants, per ear

**Parameters /Waves**	**Ear**	**Black and White Video**				**Color Video**				**p-value**
		**Mean**	**SD**	**LCI**	**UCI**	**Mean**	**SD**	**LCI**	**UCI**	
**Latencies (ms)**										
P1	RE	63.30	7.35	60.67	65.93	77.03	18.76	70.02	84.03	0.002*
N1		102.02	7.38	99.38	104.66	102.00	17.90	95.32	108.69	0.596
P2		135.87	15.42	130.35	141.38	141.18	11.62	136.83	145.52	0.000*
P1	LE	62.86	6.15	60.56	65.16	78.32	17.47	71.79	84.85	0.000*
N1		100.74	9.28	97.28	104.21	104.96	19.89	97.53	112.39	0.870
P2		133.12	19.25	125.80	140.45	139.34	22.43	119.71	136.46	0.031*
**P1-N1 Interlatency Interval (ms)**	RE	38.71	10.05	34.96	42.47	24.97	13.53	19.91	30.02	0.000*
	LE	37.88	10.28	34.04	41.72	26.64	11.54	22.51	30.77	0.000*
**N1-P2 Interlatency Interval (ms)**	RE	33.85	16.29	28.02	39.68	39.17	23.00	30.58	47.76	0.692
	LE	32.38	18.76	25.67	39.09	34.38	13.21	29,65	39,11	0.000*
**Amplitudes (µV)**										
P1	RE	2.69	1.05	2.29	3.08	2.03	1.53	1.46	2.61	0.013*
N1		-1.16	1.04	-1.55	-0.77	-1.28	1.15	-1.72	-0.85	0.820
P2		3.26	1.07	2.86	3.66	4.48	0.87	4.15	4.80	0.016*
P1	LE	2.59	1.44	2.05	3.13	2.27	1.62	1.66	2.87	0.187
N1		-0.76	1.07	-1.16	-0.36	-1.28	0.92	-1.63	-0.94	0.266
P2		3.46	0.84	3.16	3.76	3.69	0.83	3.38	4.00	0.048^*^

* , statistical difference – paired samples t-test

**Caption:** SD, standard deviation; CI, confidence interval; ms, milliseconds; RE, right ear; LE, left ear

Table 2 shows a summary of the comparisons between latency, amplitude and interlatency interval parameters of the CAEP traces collected during the presentation of the videos in black and white and color formats, in the right and left ears.

**Table 2:** Comparison between the parameters collected during the presentation of the black and white and color videos

**Ear**	**Registration Mode**	**Latency**			**Amplitude**			**Interlatency Intervals**	
		**P1**	**N1**	**P2**	**P1**	**N1**	**P2**	**P1-N1**	**N1-P2**
**RE**	Black and white	<	=	<	>	=	<	>	=
	Color	>	=	>	<	=	>	<	=
**LE**	Black and white	<	=	<	=	=	<	>	<
	Color	>	=	>	=	=	>	<	>

**Caption:** RE, right ear; LE, left ear; > larger; < smaller; = equal

Statistical analysis demonstrated differences between the analyzed parameters when comparing the traces recorded during the presentation of the video in black and white and in color. In the right ear, the P1 (p=0.002) and P2 (p=0.000) components presented higher latency values for the recordings collected during the presentation of the video in color. The P1 amplitude was lower during the presentation of the video in color (p=0.013), and the P2 amplitude was higher during the presentation of the video in color (p=0.016). In the left ear, the P1 (p=0.000) and P2 (p=0.031) latencies were also longer during the presentation of the video in color. For the amplitudes, in the left ear, the P2 component was higher during the presentation of the video in color (p=0.048).

The analysis of the interlatency intervals demonstrated that the P1-N1 interval was shorter for the recordings collected during the presentation of the color video, in the right (p=0.000) and left (p=0.000) ears. The N1-P2 interval was longer for the recording collected in the color video in the left ear (p=0.000).

When comparing the right and left ears, there was no statistically significant difference for the studied sample (p>0.05).

Table 3 shows the comparison of the variances of latencies and amplitudes between the black and white and color conditions, in both ears.

**Table 3:** Comparison of latency and amplitude variances between the black and white and color conditions, in both ears

**Standard Deviation**				
**Comparisons**	**Black and white**	**Color**	**Levene Statistics**	**p-value**
RIGHT EAR				
Latencies				
P1	7.346	18.903	18.085	0.000*
N1	7.513	19.884	18.268	0.000*
P2	18.903	11.468	4.986	0.029*
Amplitudes				
P1	1.055	1.537	1.705	0.197
N1	1.013	1.162	0.464	0.498
P2	1.539	1.999	1.765	0.173
LEFT EAR				
Latencies				
P1	8.043	17.603	12.803	0.001*
N1	10.264	19.997	8.211	0.006^*^
P2	23.25	23.158	0.171	0.681
Amplitudes				
P1	1.403	1.626	0.856	0.359
N1	1.109	0.945	0.796	0.376
P2	1.728	1.983	0.902	0.349

* , statistical difference - Levene's test

The results indicated significant differences for the comparisons of P1, N1 and P2 latencies in the right ear. In the left ear, the variances were smaller only for P1 and N1 latencies. There were no significant differences between the variances of the amplitudes.

## DISCUSSION

This discussion addresses the findings of a study that investigated the morphological differences in the CAEP traces when using black and white and color distractor videos. In this context, the study is innovative in analyzing the impact of visual characteristics of stimuli on the cortical auditory response, and the findings provide new insights into the interaction between visual and auditory stimuli in cortical processing. Detailed comparisons of latencies, interlatency intervals, and amplitudes in CAEP components between the two video conditions are explored to clarify how visual variations can influence the neural mechanisms underlying auditory perception.

In the present study, P1 and P2 presented longer latencies for the traces recorded during the presentation of the color video in both ears. P1 generally appears between 50 and 80 ms after the presentation of the acoustic stimulus and reflects the activity of the thalamocortical circuit^(22)^. Throughout the development of the child until adulthood, changes in the latency and amplitude of this wave are observed, simultaneously with the increase in myelination and synaptic efficiency^(23)^.

The P2 component is widely recognized in the literature as closely associated with the auditory discrimination process^(7)^. While there is no consensus on its neural generators, it is believed that both primary and secondary auditory cortex areas, along with the reticular formation, are influenced by this component^(24)^. The latency values of this component are influenced by age, electrode position and the state of attention and alertness of the patient being assessed^(25)^.

These findings can be attributed to the increased cognitive engagement and sensory processing demands presented by more complex visual stimuli, such as color videos. Color is a visual element that can increase stimulus salience and require additional neural processing, leading to an increased allocation of attentional resources to the visual stimulus at the expense of the auditory stimulus^(26)^.

Both the P1 and P2 waves are associated with early and mid-processing of auditory stimuli in the cortex, but their latencies can be modulated by attentional and sensory integration processes. The presentation of a color video results in enhanced processing of visual features such as hue, saturation, and brightness, which may involve not only the primary visual cortex but also associated cortical areas^(27)^. This more elaborate visual processing may compete for cognitive and attentional resources that would otherwise be available for auditory processing, resulting in prolonged latencies for the CAEP P1 and P2 components.

Furthermore, the increased sensory complexity and activation of associative neural pathways due to color visual stimuli may induce a top-down modulation of selective attention mechanisms, which affect auditory response timing^(28)^. This suggests that the simultaneous processing of more complex visual stimuli (such as color videos) may interfere with cortical auditory processing efficiency and speed, resulting in a delay in the P1 and P2 latency response.

A study using visual and auditory evoked potentials, which aimed to analyze the N1 and P2 component amplitudes and latencies during the simultaneous presentation of visual and auditory stimuli (bimodal stimulation), compared to the presentation of only one modality of visual or auditory stimulus (unimodal stimulation). The findings of the study showed the presence of longer latencies for bimodal stimulation, which, according to the authors, indicates that the concomitant presentation of visual and auditory stimuli may require more elaborate processing of sensory information, since the brain needs to integrate stimuli from different modalities (visual and auditory) simultaneously^(29)^.

A study conducted with five healthy individuals investigated specific changes in brain activity by analyzing differences in electroencephalographic waves after exposure to color and black and white visual stimuli. The authors concluded that there is greater brain activity during the presentation of color stimuli^(30)^. This finding may explain the longer latencies in the P1 and P2 components observed in the recording collected during the presentation of the color video in both ears in the present study (Table 1).

The analysis of the interlatency intervals showed a difference for the P1-N1 interval in both ears with shorter intervals in the recorded traces during the color video presentation. Since this interval corresponds to the time elapsed between the positive peak (P1) and the negative peak (N1), this result can be explained by the longer latencies of the P1 waves during the color video presentation, which, in turn, decreases the time difference between the appearance of the P1 and N1 waves in the color recording in both ears.

In contrast, the N1-P2 interlatency interval was longer for the color video presentation in the left ear, which can be explained by the prolonged latency of the P2 wave in this ear (see Table 1).

For the parameter amplitude, in turn, P1 in the right ear showed a statistical difference, with a decrease in the color video presentation. P2, in both ears, showed an increased amplitude in the color video presentation. P1 is an early component of the CAEP, associated with the initial processing of auditory stimuli in the primary auditory cortex, and its amplitude can be modulated by sensory processing factors^(31)^. Coding refers to the initial process by which the peripheral and central auditory system converts sounds into neural signals that can be interpreted by the brain. This involves the detection of fundamental characteristics of the sound, such as frequency (pitch), intensity (volume) and duration^(32)^. The P2 component is often associated with the process of auditory discrimination^(33)^. Increased amplitudes for this component may reflect greater recruitment of neural resources or greater effort to discriminate the speech stimuli /ba/ and /da/.

The presentation of a color video provides a richer array of visual information which requires increased cognitive processing and visual attention. This increase in visual cognitive load may lead to a redistribution of attentional resources, prioritizing the processing of visual stimuli over auditory stimuli. As a result, fewer neural resources may be available for auditory processing. These findings suggest that the sensory complexity of visual stimuli, such as color videos, may interfere with early auditory processing by reducing the allocation of neural resources to the auditory stimulus, resulting in a lower P1 wave amplitude.

Therefore, this decrease in P1 amplitude, the component responsible for encoding the auditory stimulus, in the right ear for color videos, can be reflected from the following analysis: as encoding is considered the most basic skill in auditory processing, as it is the first step for the brain to recognize, interpret and respond to sounds, during the “competition” between visual stimuli from the color video and the auditory stimuli, the system allocated fewer neural resources to the skill that is more basic, especially in adults (coding).

However, the higher P2 amplitudes in the right and left ears for the color videos may reflect the greater effort in discrimination, a skill considered more specialized as it requires more complex cortical processing to recognize patterns, identify subtle differences, and make decisions based on auditory stimuli. During the “competition” between visual processing and auditory processing, the system allocated more neural resources to the more advanced skill (discrimination).

A study conducted with 52 participants analyzed the detection of auditory stimuli during the presentation of visual stimuli. The visual stimuli consisted of twenty dots that changed color and were presented concomitantly with the presentation of a background noise that varied in intensity. The participants signaled their perception of these stimuli, while the images were exposed. The findings of this study suggest that visual stimulus affects the auditory perception of sounds, indicating that visual attention can compete with auditory attention^(34)^, which, ultimately, presents an important reflection on the studied topic.

Researchers investigated how different colors of visual stimuli affect steady-state visual evoked potentials (SSVEP). The authors conducted experiments using stimuli of various colors and analyzed how these colors influence the characteristics of SSVEP by analyzing amplitudes and latencies. The results showed that the color of the stimuli has a significant impact on the evoked responses, with variations in amplitudes depending on the presented color^(14)^.

Regarding the comparison of the variances of latencies and amplitudes between the different distractor videos, the results of this study indicate that there are significant differences in the variability in the P1-N1-P2 complex latencies in the right ear, and in P1 and N1 latencies in the left ear. As observed, variability was reduced during the presentation of the black and white video. These differences suggest that color stimuli may influence auditory cortical temporal processing, especially concerning the auditory encoding and decoding responses, thereby increasing the response variability^(35)^.

Previous studies indicate that visual stimuli can modulate activity in the auditory cortex, suggesting a cross-talk between the visual and auditory systems. Researchers reported that visual stimuli can modulate local field potentials in the auditory cortex, although no high-frequency activity related to active visual processing was observed in the auditory cortex. This reinforces the hypothesis that the visual system may influence auditory processing, but not necessarily at the level of generalized neuronal excitation^(36)^.

Additionally, other authors explored the impact of interactions from different sensory modalities on sensory processing, suggesting that auditory stimuli may reinforce visual memory through multisensory integration mechanisms^(37)^. These findings provide support for the visual modulation model of auditory processing, as seen in the present study, where modulation occurs in CAEP latencies but does not affect amplitude. Researchers also proposed that multisensory contributions may influence unisensory processing, demonstrating how visual stimuli can modulate auditory processing in early sensory areas, suggesting a possible modulation of the phase of neural oscillations^(38)^.

This study presents some limitations that should be considered. Although only participants without visual complaints were selected, no formal screening or documentation of visual acuity was performed. This decision was based on the study’s primary objective, which was to examine how different visual distractor formats (colored versus black-and-white) influence CAEP morphology, rather than to assess participants’ visual function. However, because visual acuity can affect the processing of visual stimuli and potentially modulate attentional engagement, the lack of this screening constitutes a methodological limitation and may introduce variability in the results.

The videos used as visual distractors were not objectively equalized for luminance, brightness, or contrast. This choice was aligned with the ecological purpose of the study, which aimed to compare the effect of typical colored versus black-and-white distractors as they are commonly used in clinical CAEP recordings. Both videos were extracted from the same source, matched in duration, and presented at the same size and distance under identical ambient lighting and equipment conditions, which helped reduce unwanted variability related to the presentation setting. However, we acknowledge that luminance- or contrast-related differences can influence visual load, attentional engagement, and consequently cortical responses. The absence of objective measurement or equalization of these parameters represents a methodological limitation, and future studies should incorporate standardized luminance and contrast calibration to more accurately isolate which visual components contribute to CAEP variability.

This study did not include objective or subjective measures of attentional state or engagement during the recording. Participants were instructed to remain attentive to the visual stimuli, and the examiner monitored their behavior throughout the session to ensure compliance with this instruction. However, because attentional fluctuations can influence cortical responses, the absence of a formal measure of attention represents a limitation of the study and may introduce variability between conditions. Future studies should incorporate behavioral or physiological metrics to better quantify participants’ attentional state during CAEP acquisition.

Therefore, future studies are needed to further explore these effects by incorporating objective measures of attention, controlling visual parameters and documenting individual participant characteristics in order to clarify the mechanisms that modulate the interaction between visual stimuli and auditory processing. In doing so, it will be possible to advance the development of more standardized and sensitive protocols, thereby contributing to the accuracy of electrophysiological assessments in both clinical and experimental contexts.

## CONCLUSION

The use of color video during the CAEP recording interferes with its trace morphology, which was proven by the longer latencies of the P1 and P2 waves in both ears, and by the lower amplitude of the P1 wave in the right ear. For the P2 wave, however, higher amplitudes were found in the right and left ears, which suggests greater allocation of neural resources for the processing of more specialized skills, such as auditory discrimination.

Furthermore, the findings suggest that color visual stimuli mainly affect CAEP variability, particularly in the P1 and N1 waves, but without significantly affecting the variability of amplitude responses**.** Therefore, the use of black and white videos should be recommended as a distractor resource during CAEP testing with speech stimuli in adults.
